# Prevalence of Hypothyroidism in Children and Adolescents of Fars Province (Southern Iran): A Nationwide Prescription Study, 2018–2019

**DOI:** 10.34172/aim.2022.69

**Published:** 2022-07-01

**Authors:** Alireza Mirahmadizadeh, Razieh Bahadori, Gholamhossein Ranjbar Omrani, Forough Saki, Mehrab Sayadi

**Affiliations:** ^1^Non-communicable Diseases Research Center, Shiraz University of Medical Sciences, Shiraz, Iran; ^2^Shiraz University of Medical Sciences, Shiraz, Iran; ^3^Shiraz Endocrinology and Metabolism Research Center, Shiraz University of Medical Sciences, Shiraz, Iran; ^4^Cardiovascular Research Center, Shiraz University of Medical Sciences, Shiraz, Iran

**Keywords:** Adolescents, Children, Hypothyroidism, Iran

## Abstract

**Background:**

Hypothyroidism is the most common hormonal deficiency worldwide; however, there is limited data about its prevalence in the children and adolescents of the Middle East.

**Methods:**

The prevalence of hypothyroidism were calculated by dividing the number of patients purchasing levothyroxine in 1397 Solar-Hijri year (Correlate with March 2018-February 2019) by the population at risk (per 10000 persons). Data were collected from the Iran health insurance organization registration records and family physician databases of health vice-chancellor of Shiraz University of Medical Sciences.

**Results:**

The present study shows that the prevalence of levothyroxine treated population aged under 18 years is 13 in 10000 in the Fars province and it is more common in females (17 in 10000 in females versus 9 in 10000 in males). This study also revealed that the prevalence of hypothyroidism was different in various age groups and increased in older children and adolescents after pubertal ages. Also, an increase in the female: male ratio of prevalence was more obvious during and after puberty.

**Conclusion:**

Our study showed that the prevalence of congenital hypothyroidism was 3/10000 in southern Iran. Also, the prevalence of hypothyroidism in children and adolescents was totally 13/10000 population, and this prevalence increased in older age and female gender. This prevalence was close to the data from iodine sufficient areas in Europe and the United States.

## Introduction

 In hypothyroidism, the thyroid gland fails to produce or secrete the demanded amounts of thyroid hormones sufficient for body metabolisms.^1^ It is the most common hormonal deficiency, classified as primary hypothyroidism in which the defect is at the level of the thyroid gland, and the secondary type in which insufficient thyroid-stimulating hormone (TSH) function causes thyroid hormone deficiency. Levothyroxine is the treatment of choice in both groups.^1^

 Early diagnosis and treatment of hypothyroidism during childhood is important because it prevents future mental retardation and growth impairment.^2^ Hypothyroidism during childhood is classified as the congenital type which is present during the first six months of life and the acquired type in which symptoms present mostly after the age of 6 months.^3^ The prevalence of congenital hypothyroidism is well described because of programmed neonatal screening in many countries; however, the prevalence of acquired hypothyroidism in the young ages has not been well studied, particularly in recent years.^4^ The neonatal disease screening program in Iran began in October 2004.^5^ After that time, many investigations were done to estimate the prevalence of congenital hypothyroidism and revealed that it occurred at about 0.15%, 0.2% and 0.3-0.34% in the west, north, and center of Iran, respectively.^3,6-8^ These data are comparable to the incidence of congenital hypothyroidism worldwide (1:3000–1:4000).^9^

 Among the patients diagnosed with congenital hypothyroidism in Iran, about 60% were confirmed to have permanent hypothyroidism.^10^ The rate of permanent cases of congenital hypothyroidism varied worldwide in the range of 38-62%.^11^ Different factors such as iodine deficiency, iodine overload, trans-placental crossing of thyrotropin (TSH) receptor blocking antibodies, production of thyroid autoantibodies, using anti-thyroid drug or iodine-containing antiseptics ingestion during pregnancy, maternal consumption of goitrogens (foods and drugs), very low birth weight and prematurity, and some gene mutations could cause differences in the prevalence of permanent congenital hypothyroidism.^12-14^

 Although there are many investigations about the prevalence of congenital hypothyroidism, there have been few studies on the prevalence of hypothyroidism during childhood and adolescence worldwide. These studies were limited to investigations that include the general population in all age groups in Europe.^1,15-20^ In addition, there are few studies about hypothyroidism in children just including small selected aged groups; it was shown that the prevalence of hypothyroidism was about 0.04-0.06% in children and adolescents.^21-23^ An interesting study in the United Kingdom evaluated the prevalence and etiology of hypothyroidism in the young age groups.^4^ Their method was prescription-based and included young patients in Scotland who had received two or more prescriptions for thyroxine from January 1993 to December 1995, in those aged less than 22 years.^4^ Because thyroxine should be prescribed for young people only if they are clinically and biochemically diagnosed with hypothyroidism, and these drugs were not used to treat other diseases, prescription of the drug can be used as a surrogate marker of hypothyroidism. They showed that the overall prevalence of hypothyroidism in young people under 22 years is 0.135%, and it is at least twice the previous estimates.^4^ Due to lack of sufficient data about the prevalence of hypothyroidism in children and adolescents under 18 years of age worldwide, the present study aimed to determine the prevalence of patients who had received at least two times thyroxine prescriptions in the population of children and adolescents aged under 18 years in the Fars province (southern Iran) during March 2018- February 2019.

## Materials and Methods

 Iran has a national health insurance program that covers all residents living in this country expect those groups who have some special institutional insurance. All the information is saved with a unique national personal code used to identify each insured person. This code identifies the holder and shows his/her birth date, gender, and birthplace. Also, there is a unique health data service in Shiraz University of Medical Science that has gathered all the medical product prescriptions by physicians that saved the name of drugs, name of physician, and national code of patients in the Fars province, southern Iran.

 Levothyroxine is the only treatment available in Iran; hence, in our country the treatment of hypothyroidism was solely done using levothyroxine sodium tables (0.1 mg, 50 µg and 25 µg), and it is available by prescription only. The prescriptions are valid up to 3-6 months from the prescription day, and children receive regular assessment of thyroid function every 3-6 months. Hence, patients with hypothyroidism who need daily drug consumption need prescriptions at least twice a year.

###  Data Collection

 Information about the drug presentations in the Fars province and data of patients are gathered by the National Health Insurance Scheme and Shiraz University of Medical Science health vice-chancellor, established during March 2018-February 2019 (i.e. 1397 Hijri year). The Fars province is located in southern Iran and has a population of 4.9 million, and about 2 700 000 are under coverage of the Iran health insurance organization. These include people living in both urban and rural areas, without any distinction in terms of income, job, city or education level. Hypothyroidism cases were confirmed by the family physician database who are residents of the Fars province. Data on age and gender were matched with the drug prescription using V-lookup (vertical lookup) function in Microsoft excel software.

###  Statistical Analysis

 The prevalence of hypothyroidism was calculated by dividing the number of patients purchasing levothyroxine in 1397 Hijri year (corresponding to March 2018-February 2019) by the population at risk (per 10 000 persons). All prevalence rates calculated including cases (numerator) and population (denominator) were exclusively from the mentioned insurance.

 A Poisson distribution was used to calculate the 95% confidence interval (CI) for the prevalence. The statistical significance of the effect of gender was quantified by calculating the prevalence with a 95% CI. *P* values less than 0.05 were considered as statistically significant. Statistical analysis was done using the SPSS software 21.

## Results

**Figure 1:**
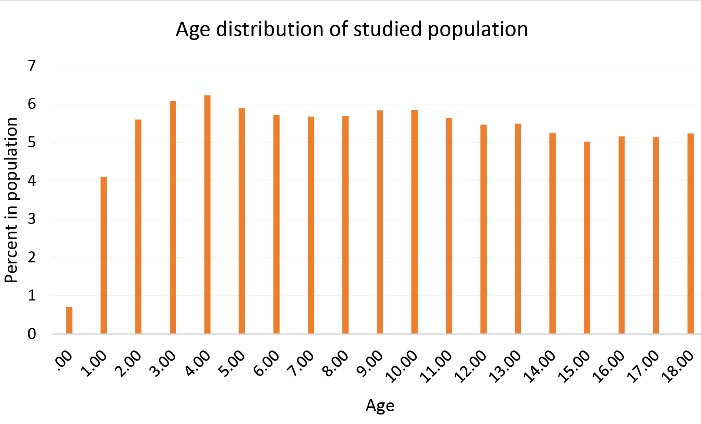
Age Distribution of the Study Population

 During March 2018-February 2019, a total of 265,605 probably at risk people under 18 years of age were included in this study who were under coverage of the National Health Insurance system in the Fars province, of whom 128,382 were female (48.3%). The age distribution of this population is shown in Figure 1.

 Totally, 9339 tablets of levothyroxine 50 µg and 72 873 tablets 100 µg were prescribed for 1315 cases who consumed at least one dose of levothyroxine per year. In the studied population, 347 children who received at least 2 times levothyroxine prescription (0.13%), were labeled as having hypothyroidism. Of these patients, 219 (63%) were female and 128 (37%) were male. The female-to-male ratio of childhood hypothyroidism was 1.7:1.

 In addition, for further evaluation and comparison of our data with other similar studies, we decided to categorize our patients based on age (0-3, 4-7, 8-11, 12-15, 16-18 years).

Table 1 displays the summary of the number of patients and prevalence (per 100 000) of levothyroxine treated hypothyroidism which was compared by age and gender. As the table shows, hypothyroidism is more common in females except for those under 3 years of age, and the prevalence increases with age. To control the confounders effect, Poisson regression was done and after adjustment for age and gender, the prevalence of hypothyroidism in females was higher than males (*P* < 0.001) and increased significantly with age (*P* < 0.001).

**Table 1:** Number of Cases and Prevalence (Per 100 000) of Levothyroxine Treated Hypothyroidism by Gender and Age Groups

**Age Group (y)**	**Female**		**Prevalence (95% CI)**	**Male**		**Prevalence (95% CI)**	**Gender Prevalence Ratio (F/M)** ^*^
	**Population **	**Cases**		**Population **	**Cases**		
0–3	21,142	7	33.11 (8.58–57.64)	22,727	7	30.8 (8.00–53.73)	1.075
4–7	30,094	24	79.75 (47.75–111.40)	32,279	20	61.96 (34.81–89.11)	1.287
8–11	29,557	52	175.93 (127.93–223.33)	31,470	29	92.15 (58.62–125.67)	1.909
12–15	27,086	58	214.13 (159.08–269.18)	29,235	37	126.56 (85.80–167.32)	1.691
16 +	20,393	78	382.48 (297.75–467.21)	21,565	35	162.3 (108.57–216.02)	2.356
Total	128,272	219	170.58 (148.01–193.15)	137,276	128	93.27 (77.12–109.43)	1.828

*P > 0.05, by Chi square test.

## Discussion

 The present study shows that the prevalence of levothyroxine treated population aged under 18 years is 13 in 10 000 in the Fars province and it is more common in females (17 in 10 000 in females versus 9 in 10 000 in males). This study also revealed that prevalence of hypothyroidism was different in various age groups and increased in older children and adolescence after pubertal ages. Also, the increase in the female-to-male ratio of prevalence was more obvious during and after puberty.

 A seven-year study on the prevalence of congenial hypothyroidism in northern Iran showed that the prevalence was about 1 per 491 live births in 2007, which was much higher than the figures reported in other countries in Asia,^24^ Europe^25^ and America.^26^ However, this prevalence was close to the data from Turkey^27^ (1:650 births) and Pakistan.^28^ Another study in the central part of Iran revealed that the prevalence of congenital hypothyroidism in newborn screening test was 1:307.^8^ However, Hashemipour et al showed that the prevalence rates of transient and permanent congenital hypothyroidism in Isfahan (in central part of Iran) were about 1:1114 and 1:478 live births, respectively.^10^ Taee et al showed that the prevalence of congenital hypothyroidism in Khorramabad (located in western Iran) was 0.143%.^6^ In the present study, we found that the prevalence of levothyroxine-treated children in the Fars province located in southern Iran was about 3:10 000 (0.03%), which is close to the results of a previous study in southern Iran.^29^ In that previous study, Karamizadeh et al reported that the prevalence of congenital hypothyroidism was 1:1465 and the most common cause might have been dyshormonogenesis.^29^ Our data from southern Iran showed that this prevalence was close to previous reports in the Fars province and in the United States (1:3378).^30^ Factors such as iodine sufficiency, genetic variations, and disparities in TSH levels used in previous reports to define hypothyroidism could cause a difference in prevalence among various areas.^4,30^ In spite of several investigations on the prevalence of congenital hypothyroidism, there is limited data about the prevalence of hypothyroidism during childhood and adolescence in Iran. Ingoe et alstudied the prevalence of treated hypothyroidism in North-East England.^17^ They showed that the prevalence of hypothyroidism in their population increased with age and reached 14% in people over 90 years.^17^ Serna Arnaiz et al showed that the prevalence of hypothyroidism was 3.6% in the age group under 15 years in Spain in 2001.^18^ Greggio et al showed that the prevalence of treated subclinical hypothyroidism was 0.2 per 1000 children during 2001-2014.^19^ Hunter et al reported that the prevalence of clinical hypothyroidism in young people under 22 years of age was 135:100 000 in Scotland and increased with age.^4^ Another study in Finland showed that the prevalence of hypothyroidism was 0.29% and 0.12% in females and males aged under 19 years.^15^ The present study showed that prevalence of hypothyroidism was 13:10000 population (0.13%) which is close to the data in iodine sufficient areas,^15^ and this prevalence increased with age. This prevalence was 0.07% in the age of 4–7 years and increased to 0.13%, 0.17% and 0.27% in ages 8–11, 12–15 and 16–18 years, respectively. Our data also revealed that the prevalence of hypothyroidism in the age group of 0-3 years was similar in both genders; however, as age increased, the female-to-male ratio rose, in a pattern of 1.3, 2, 1.6, and 3.6 in age groups of 4–7, 8–11, 12–15 and 16-18, respectively. There is a mild to severe iodine deficiency disorder in Middle East countries, due to lack of effective iodine supplementation. However, Iran has achieved the goal of universal salt iodization.^31,32^ Hence, the prevalence of hypothyroidism in children and adolescents in our study is close to the figures from iodine sufficient areas.^15,33^ A similar pattern of increasing prevalence of hypothyroidism was seen in Finland^15^ and Sweden.^34^ In addition, previous reports from Spain, Italy, the United Kingdom, and Finland also showed that hypothyroidism was more common in females.^4,15,17-20^

 In spite of several important points of this study which is the first one in the Middle East to investigate the prevalence of hypothyroidism in children and adolescents determined by thyroid hormone consumption, it had some limitations. First, we could not differentiate the patients with subclinical hypothyroidism because we did not have access to the laboratory data of the patients. The second is that we could not evaluate the levothyroxine dosage during therapy and could not investigate the associated autoimmune disorders. The third is that although hypothyroidism cases were confirmed by the family physician database who are residents of the Fars province, and we assumed that they would buy their drugs in the Fars province, there is a possibility to purchase the medication outside the province. Future cohort studies are recommended to remove these weak points.

 In conclusion, our study showed that the prevalence of congenital hypothyroidism was 3/10 000 in southern Iran. Also, the prevalence of hypothyroidism in children and adolescents was totally 13/10 000 population, and this prevalence increased in older age and female gender. This prevalence was close to the data from iodine sufficient area in Europe and the United States. Future cohort studies are recommended to determine the appropriate levothyroxine dosage and find out the prevalence of subclinical forms.

## References

[1] Giorda CB, Carnà P, Romeo F, Costa G, Tartaglino B, Gnavi R (2017). Prevalence, incidence and associated comorbidities of treated hypothyroidism: an update from a European population. Eur J Endocrinol.

[2] Rose SR, Brown RS, Foley T, Kaplowitz PB, Kaye CI, Sundararajan S (2006). Update of newborn screening and therapy for congenital hypothyroidism. Pediatrics.

[3] Hashemipour M, Ghasemi M, Hovsepian S, Heiydari K, Sajadi A, Hadian R (2013). Prevalence of permanent congenital hypothyroidism in Isfahan-Iran. Int J Prev Med.

[4] Hunter I, Greene SA, MacDonald TM, Morris AD (2000). Prevalence and aetiology of hypothyroidism in the young. Arch Dis Child.

[5] Yarahmadi S, Azhang N, Nikkhoo B, Rahmani K (2020). A success story: review of the implementation and achievements of the National Newborn Screening Program for congenital hypothyroidism in Iran. Int J Endocrinol Metab.

[6] Taee N, Faraji Goodarzi M, Safdari M, Bajelan A (2019). A 10-year prevalence of congenital hypothyroidism in Khorramabad (Urban Western Iran). Mol Genet Genomic Med.

[7] Beheshti Z, Rezaei R, Alipour A, Kosarian M, Saatsaz S (2018). A 7-year study on the prevalence of congenital hypothyroidism in northern Iran. Electron Physician.

[8] Dorreh F, Yousefi Chaijan P, Javaheri J, Zeinalzadeh AH (2014). Epidemiology of congenital hypothyroidism in Markazi province, Iran. J Clin Res Pediatr Endocrinol.

[9] Grüters A, Liesenkötter KP, Zapico M, Jenner A, Dütting C, Pfeiffer E (1997). Results of the screening program for congenital hypothyroidism in Berlin (1978-1995). Exp Clin Endocrinol Diabetes.

[10] Hashemipour M, Hovsepian S, Kelishadi R, Iranpour R, Hadian R, Haghighi S (2009). Permanent and transient congenital hypothyroidism in Isfahan-Iran. J Med Screen.

[11] Gaudino R, Garel C, Czernichow P, Léger J (2005). Proportion of various types of thyroid disorders among newborns with congenital hypothyroidism and normally located gland: a regional cohort study. Clin Endocrinol (Oxf).

[12] Vermiglio F, Lo Presti VP, Scaffidi Argentina G, Finocchiaro MD, Gullo D, Squatrito S (1995). Maternal hypothyroxinaemia during the first half of gestation in an iodine deficient area with endemic cretinism and related disorders. Clin Endocrinol (Oxf).

[13] Ordookhani A, Pearce EN, Mirmiran P, Azizi F, Braverman LE (2007). The effect of type of delivery and povidone-iodine application at delivery on cord dried-blood-specimen thyrotropin level and the rate of hyperthyrotropinemia in mature and normal-birth-weight neonates residing in an iodine-replete area: report of Tehran province, 1998-2005. Thyroid.

[14] Olivieri A, Fazzini C, Medda E (2015). Multiple factors influencing the incidence of congenital hypothyroidism detected by neonatal screening. Horm Res Paediatr.

[15] Virta LJ, Eskelinen SI (2011). Prevalence of hypothyroidism in Finland--a nationwide prescription study. Eur J Clin Pharmacol.

[16] Bjoro T, Holmen J, Krüger O, Midthjell K, Hunstad K, Schreiner T (2000). Prevalence of thyroid disease, thyroid dysfunction and thyroid peroxidase antibodies in a large, unselected population The Health Study of Nord-Trondelag (HUNT). Eur J Endocrinol.

[17] Ingoe L, Phipps N, Armstrong G, Rajagopal A, Kamali F, Razvi S (2017). Prevalence of treated hypothyroidism in the community: analysis from general practices in North-East England with implications for the United Kingdom. Clin Endocrinol (Oxf).

[18] Serna Arnáiz MC, Galván Santiago L, Gascó Eguiluz E, Manrique Manrique M, Foix Oña MM, Martín Gracia E (2003). [Estimate of hypothyroidism prevalence in Lleida, Spain, based on thyroid hormone prescription]. Rev Esp Salud Publica.

[19] Greggio NA, Rossi E, Calabria S, Meneghin A, Gutierrez de Rubalcava J, Piccinni C (2017). Subclinical hypothyroidism in paediatric population treated with levothyroxine: a real-world study on 2001-2014 Italian administrative data. Endocr Connect.

[20] Escribano-Serrano J, Paya-Giner C, Méndez Esteban MI, Márquez-Ferrando M, Zarallo-Pérez A, Michán-Doña A (2014). [Different methods used to estimate the prevalence of hypothyroidism, Cadiz, Spain]. Rev Esp Salud Publica.

[21] Rastogi MV, LaFranchi SH (2010). Congenital hypothyroidism. Orphanet J Rare Dis.

[22] Jaksić J, Dumić M, Filipović B, Ille J, Cvijetić M, Gjurić G (1994). Thyroid diseases in a school population with thyromegaly. Arch Dis Child.

[23] Rallison ML, Dobyns BM, Meikle AW, Bishop M, Lyon JL, Stevens W (1991). Natural history of thyroid abnormalities: prevalence, incidence, and regression of thyroid diseases in adolescents and young adults. Am J Med.

[24] Gu X, Wang Z, Ye J, Han L, Qiu W (2008). Newborn screening in China: phenylketonuria, congenital hypothyroidism and expanded screening. Ann Acad Med Singap.

[25] Kumorowicz-Czoch M, Tylek-Lemanska D, Starzyk J (2011). Thyroid dysfunctions in children detected in mass screening for congenital hypothyroidism. J Pediatr Endocrinol Metab.

[26] Mitchell ML, Hsu HW, Sahai I (2011). The increased incidence of congenital hypothyroidism: fact or fancy?. Clin Endocrinol (Oxf).

[27] Dilli D, Çzbaş S, Acıcan D, Yamak N, Ertek M, Dilmen U (2013). Establishment and development of a national newborn screening programme for congenital hypothyroidism in Turkey. J Clin Res Pediatr Endocrinol.

[28] Afroze B, Humayun KN, Qadir M (2008). Newborn screening in Pakistan - lessons from a hospital-based congenital hypothyroidism screening programme. Ann Acad Med Singap.

[29] Karamizadeh Z, Saneifard H, Amirhakimi G, Karamifar H, Alavi M (2012). Evaluation of congenital hypothyroidism in fars province, iran. Iran J Pediatr.

[30] Olney RS, Grosse SD, Vogt RF Jr (2010). Prevalence of congenital hypothyroidism--current trends and future directions: workshop summary. Pediatrics.

[31] Mohammadi M, Azizi F, Hedayati M (2018). Iodine deficiency status in the WHO Eastern Mediterranean Region: a systematic review. Environ Geochem Health.

[32] Nazeri P, Mirmiran P, Mehrabi Y, Hedayati M, Delshad H, Azizi F (2010). Evaluation of iodine nutritional status in Tehran, Iran: iodine deficiency within iodine sufficiency. Thyroid.

[33] Delange F (2002). Iodine deficiency in Europe and its consequences: an update. Eur J Nucl Med Mol Imaging.

[34] Manousou S, Augustin H, Eggertsen R, Hulthén L, Filipsson Nyström H (2021). Inadequate iodine intake in lactating women in Sweden: a pilot 1-year, prospective, observational study. Acta Obstet Gynecol Scand.

